# Topical EOSSKY fullerene moisturizing and repairing cream for preventing acute radiation dermatitis in breast cancer patients undergoing radiotherapy: a randomized controlled trial

**DOI:** 10.3389/fmed.2025.1604012

**Published:** 2025-07-22

**Authors:** Qi Wang, Xiaoning Shi, Jinli Guo, Zhanjun Gu, Xinghua Dong, Yanxia Qin

**Affiliations:** ^1^College of Nursing, Shanxi Medical University, Taiyuan, Shanxi, China; ^2^Second Hospital of Shanxi Medical University, Taiyuan, Shanxi, China; ^3^Institute of High Energy Physics and National Center for Nanoscience and Technology, CAS Key Laboratory for Biomedical Effects of Nanomaterials and Nanosafety and CAS Center for Excellence in Nanoscience, Chinese Academy of Sciences, Beijing, China; ^4^Guangdong-Hong Kong-Macao Greater Bay Area National Institute of Nanotechnology Innovation and Research, Guangzhou, Guangdong, China; ^5^Guangzhou Guangna Huichuan Technology Co., Guangzhou, Guangdong, China

**Keywords:** fullerene, acute radiation dermatitis, radiotherapy, breast cancer, randomized controlled trial

## Abstract

**Background and Purpose:**

Acute radiation dermatitis (ARD) is a prevalent complication among breast cancer patients undergoing radiotherapy. Fullerene possesses broad-spectrum free radical scavenging properties, which may be beneficial in mitigating oxidative stress. This study aimed to evaluate the impact of fullerene on both the incidence and severity of ARD in breast cancer patients.

**Methods:**

This study involved 88 breast cancer patients who met the inclusion criteria. Participants were randomly assigned to either the fullerene group or the control group in a 1:1 ratio. The primary endpoint was the grading of ARD and the cumulative dose of ionizing radiation at the first occurrence of ARD. Secondary endpoints included patient-reported symptoms, such as pain and quality of life (QoL).

**Results:**

The Radiation Therapy Oncology Group (RTOG) score was significantly lower in the fullerene group than in the control group, and the cumulative dose of ionizing radiation at the first occurrence of ARD was higher. There was a statistically significant difference between the two groups (*P* < 0.05). Regarding the secondary endpoints, the pain scores exhibited a significant reduction in the fullerene group as compared with the control group (*P* < 0.05). The results of the Skindex-16 scale showed that the quality of life was better in the fullerene group than in the control group (*P* < 0.01).

**Conclusion:**

This trial indicated that fullerene could reduce the grading of ARD, delay the occurrence of ARD, alleviate patients’ symptoms, and improve the patients’ overall quality of life.

**Clinical trial registration:**

https://www.chictr.org.cn/index.html, identifier CTR2400079800.

## 1 Introduction

Cancer is a significant public health issue, with female breast cancer remaining the leading cause of both cancer incidence and cancer-related mortality among women worldwide (1). The American Cancer Society (ACS) estimates that in 2024, there were 310720 new cases of breast cancer in the United States, representing 15.5% of all new cancer diagnoses (2). Radiotherapy is used in >50% of patients with cancer, about 40% of cancer can be cured (3–5).

However, according to statistical data, acute radiation dermatitis (ARD) occurs to varying degrees in 95% of cancer patients undergoing radiotherapy and is a common complication among those receiving radiotherapy for breast, head and neck cancers (6, 7). ARD can contribute to a negative patient experience, reduce health-related quality of life, and lead to poor compliance with administered treatments (8).

In the 2023 clinical practice guidelines published by the Multinational Association of Supportive Care in Cancer (MASCC), the most promising interventions for the prevention and management of ARD include photobiomodulation therapy, Mepitel film, Hydrofilm, olive oil, oral enzyme mixtures, mometasone furoate, and betamethasone (9). A gold-standard treatment for the prevention and management of ARD has yet to be established, despite the extensive body of evidence supporting various therapeutic regimens (7).

Fang Fu Gao is a cream containing Trolamine. Trolamine is a compound similar to non-steroidal anti-inflammatory agents with concomitant antioxidant effects and has been considered a safe and tolerable ARD topical intervention (10, 11), which has been supported by multiple randomized trials and meta-analyses (12–14). Furthermore, trolamine-based emulsions have been a standard therapy for radiation dermatitis in Europe and the United States for over three decades (15).

Fullerene, known as a “free radical sponge,” is a great choice for skin radioprotection because of its broad-spectrum free radical scavenging performance, good chemical stability, and excellent biosafety (16). Currently, the research on hydroxy fullerene used in radiotherapy protection mainly focuses on basic research, and the *in vitro* and *in vivo* experiments proved that fullerene agents are effective against ARD (17). Therefore, we designed and large-scale synthesized protective ointment with fullerene as the main ingredient (EOSSKY Fullerene moisturizing and Repairing cream). This study aimed to conduct a randomized control trial to investigate the prophylactic effects of EOSSKY Fullerene moisturizing and Repairing cream versus control intervention (Fang Fu Gao, a cream containing Trolamine) for ARD in patients with breast cancer receiving radiotherapy. It also provides a new idea for the prevention of ARD and provides a reference for the application and popularization of fullerene in ARD patients.

## 2 Methods and materials

### 2.1 Study design and sample size

The design of this study was a randomized controlled trial comparing EOSSKY Fullerene moisturizing and Repairing cream with a control intervention in 88 breast cancer patients undergoing radiation therapy. The study was approved by the Ethics Committee of the Second Hospital of Shanxi Medical University (approval number: 2023, ethics number 190) and written informed consent was obtained from all participants. The CONSORT guidelines and checklist, Good Clinical Practice principles, and the Declaration of Helsinki were rigorously followed. Trial registration: Chinese Clinical Trial Registry identifier: CTR2400079800; Date of registration: 12/01/2024.

The results of our pilot study indicated that EOSSKY Fullerene moisturizing and Repairing cream has good therapeutic effects and safety. The sample size was calculated using the formula for comparing two sample rates, with α = 0.05 and β = 0.1. Considering a 15% dropout rate, the final total sample size was determined to be 88 cases, with 44 cases in the EOSSKY Fullerene moisturizing and Repairing cream group and 44 cases in the control group.

### 2.2 Patient selection

Eligibility criteria for the study participants included the following: patients diagnosed with breast cancer based on pathological examination; female patients aged between 18 and 75; surgery with modified radical or breast-conserving surgery; fully healed surgical incision; planning to receive postoperative radiation therapy; Karnofsky Performance Status (KPS) score ≥70; no severe endocrine or metabolic disease; Exclusion criteria included: receiving concurrent chemotherapy; presence of skin disease; receiving other interventions to prevent or treat ARD during treatment.

### 2.3 Radiotherapy

Both groups of patients received conformal intensity modulated radiation therapy (IMRT) in a fractionated manner, with a single dose of 2 Gy, five fractions per week up to a total dose of 50–60 Gy.

### 2.4 Randomization and blinding

Participants were recruited from the department of oncology, The Second Hospital of Shanxi Medical University. Before the study initiation, a computer-generated random allocation sequence (1–88) was produced using SPSS 26.0, which assigned random sequence numbers to either Group A (experimental group) or Group B (control group) in a 1:1 allocation ratio. The allocation results were sequentially sealed in opaque envelopes and distributed sequentially at the time of patient admission. Since the drug colors of the experimental group and the control group were significantly different, the blind method was not adopted.

### 2.5 Grouping and ARD management

In this study, patients were divided into the Fang Fu Gao group and the fullerene group. Both groups received routine health education and were provided with information about ARD before the start of the treatment. Besides, for the fullerene group and control group, patients were asked to start topical application of EOSSKY Fullerene moisturizing and Repairing cream and control intervention cream, respectively, on the area of skin being irradiated at the onset of radiotherapy, three times a day at the same dose until the treatment was over.

### 2.6 Study end points

The primary endpoint were the grading of ARD and the cumulative dose of ionizing radiation at the first occurrence of ARD. The secondary study endpoints were patient-reported symptoms, including pain, quality of life, and adverse events. ARD was graded using the North American Radiation Therapy Oncology Group (RTOG) grading criteria, skin pain was assessed using the Visual Analog Scale (VAS) assessment tool, and QoL was measured using the Skindex-16 scale. Adverse events (e.g., edema, pruritus) were assessed by a combination of daily researcher observations and patient-reported symptoms.

### 2.7 Clinical evaluation

Acute radiation dermatitis was assessed every day by the investigator throughout the treatment period, and the grading of ARD and the cumulative dose of ionizing radiation were recorded. Patients’ skin pain was evaluated weekly during treatment using the VAS assessment tool. The higher the score, the more intense the pain. The patients’ QoL was assessed using the Skindex-16 scale every week. Adverse events were assessed daily by a combination of researcher observation and patient reports.

### 2.8 Statistical analysis

The IBM SPSS Statistics 26 software was used for statistical analysis. If the continuous variables conform to a normal distribution, the mean ± standard deviation is used to describe, and the two independent sample *t*-test is used to compare continuous variables between groups. If not, the medians and quartiles would be used to describe, and the Wilcoxon Rank Sum test would be used to compare the differences between the two groups. Categorical variables are described as frequencies and percentages, and Fisher’s exact test or Chi-square test is used to compare variables between groups. *P* < 0.05 was considered the basis of statistical significance.

## 3 Results

### 3.1 Patient characteristics

**FIGURE 1:**
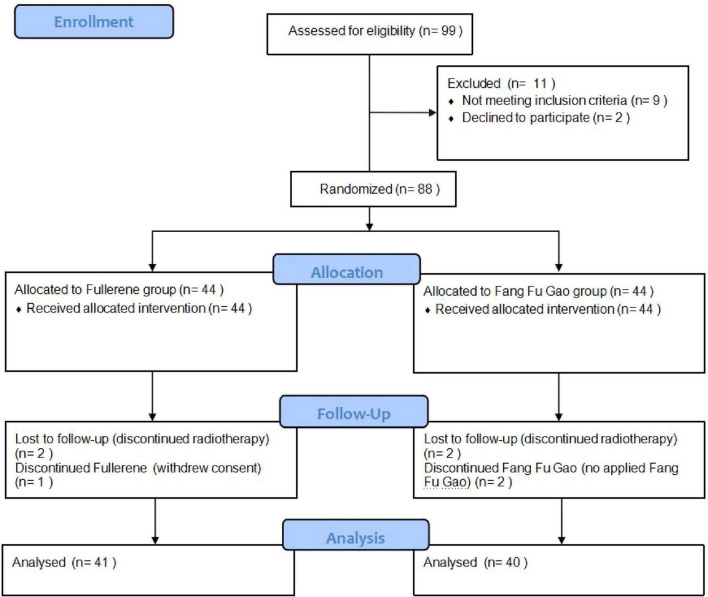
Patient flowchart.

A total of 88 patients were included in this study between May 2023 and January 2025 (Figure 1). Forty-four patients received EOSSKY Fullerene moisturizing and Repairing cream, while the other forty-four patients received Fang Fu Gao as the control group. After randomization, one patient in the fullerene group withdrew consent, and two patients failed to complete radiotherapy. Two patients in the control intervention group were excluded due to incomplete radiotherapy, and two patients did not use Fang Fu Gao. As a result, a total of 81 eligible patients were ultimately included. The baseline characteristics of the two groups had no statistically significant differences (*P* > 0.05; Table 1).

**TABLE 1:** Comparison of baseline characteristics comparison between two groups.

	Fullerene group (*n* = 41)		Fang Fu Gao group (*n* = 40)		*P*-value
	Mean (SD)		Mean (SD)		
Age (years)	54.28 (8.89)		53.61 (10.65)		0.761
	***n***	**%**	***n***	**%**	
Educational level					0.711
Primary school and below	17	41.46	16	40.00	
Junior high school	19	46.34	17	42.50	
Senior high school and above	5	12.20	7	17.50	
Surgery method					0.221
Breast conserving surgery	22	53.66	16	40.00	
Modified radical surgery	19	46.34	24	60.00	
Planned total radiation dose					0.221
50 Gy	22	53.66	16	40.00	
60 Gy	19	46.34	24	60.00	
Adjuvant endocrine therapy					0.615
Yes	9	21.95	7	17.50	
No	32	78.05	33	82.50	
Adjuvant targeted therapy					0.753
Yes	5	12.20	4	10.00	
No	36	87.80	36	90.00	

### 3.2 Efficacy

According to the RTOG classification, the ARD grading at the end of radiotherapy was significantly better in the fullerene group compared with the Fang Fu Gao group (Table 2). There was a statistically significant difference between the two groups (*P* < 0.05).

**TABLE 2:** Acute radiation dermatitis (ARD) grading.

	ARD grade 0	ARD grade 1	ARD grade 2	ARD grade 3	ARD grade 4	*P*-value
	*n* (%)	*n* (%)	*n* (%)	*n* (%)	*n* (%)	
Fullerene group (*n* = 41)	11 (26.83)	28 (68.29)	2 (4.88)	0	0	0.002
Fang Fu Gao group (*n* = 40)	3 (7.50)	25 (62.50)	12 (30.00)	0	0	

The cumulative dose of ionizing radiation at the first occurrence of ARD was higher in the fullerene group than in the control group (Table 3). In other words, the use of fullerene delayed the incidence of ARD in the intervention group. This was confirmed by comparing the time to the first ARD appearance in the two groups (Table 4). There was a statistically significant difference between the two groups (*P* < 0.01).

**TABLE 3:** The cumulative dose of ionizing radiation at the first occurrence of ARD.

Grade	Fullerene group (Gy)		Fang Fu Gao group (Gy)		*P*-value
	*n*	Mean ± SD	*n*	Mean ± SD	
0	11	/	3	/	/
1	28	42.00 ± 5.02	25	25.28 ± 4.72	<0.001
2	2	57.00 ± 1.41	12	37.33 ± 2.46	<0.001
3	0	/	0	/	/
4	0	/	0	/	/

**TABLE 4:** Number of days to first ARD appearance.

	First ARD appearance (days)		*P*-value
	Median	(P_25_, P_75_)	
Fullerene group (*n* = 41)	29	(25.75, 31.00)	<0.001
Fang Fu Gao group (*n* = 40)	18	(14.50, 19.50)	

After evaluation of adverse events, no adverse events unrelated to the intended use of the medication were identified.

The pain scores in the fullerene group were consistently lower than those in the control group (Table 5). There was a significant difference in pain between the fullerene and control groups starting from the third week (*P* < 0.05).

**TABLE 5:** Comparison of pain scores between the two groups.

	Pain scores [Mean (SD)]					
	Week 1	Week 2	Week 3	Week 4	Week 5	Week 6
Fullerene group (*n* = 41)	0	0.07 (0.26)	0.39 (0.63)	0.95 (0.44)	1.29 (0.56)	1.85 (0.61)
Fang Fu Gao group (*n* = 40)	0	0.20 (0.41)	0.78 (0.66)	1.30 (0.69)	2.08 (0.73)	2.68 (0.53)
*P*-value	/	0.098	0.009	0.009	<0.001	<0.001

The results of the Skindex-16 scale scores showed that the quality of life was better in the fullerene group than in the control group after radiation therapy (Table 6). There was a statistically significant difference between the two groups (*P* < 0.001).

**TABLE 6:** Comparison of Skindex-16 scale scores between the fullerene group and the control group.

	Skindex-16 scale scores [Mean (SD)]		*P*-value
	Fullerene group (*n* = 41)	Fang Fu Gao group (*n* = 40)	
Symptoms score	21.03 (2.81)	15.17 (2.18)	<0.001
Emotion score	16.38 (4.36)	10.00 (2.42)	<0.001
Functioning score	12.75 (2.70)	8.39 (1.90)	<0.001
Total score	50.15 (7.86)	33.56 (4.91)	<0.001

## 4 Discussion

Because the skin is the first organ exposed to radiation, ARD is the most common profound side effect of breast cancer patients undergoing radiotherapy (18). As cumulative radiation doses are received, skin damage becomes increasingly apparent, manifesting as erythema, dryness, peeling, folliculitis, itching, hyperpigmentation, pain, and mucosal ulceration (19). The pathophysiology of ARD involves X-rays causing DNA damage through direct interactions with DNA, as well as producing excessive reactive oxygen species (ROS). Additionally, indirect radioactive reactions with water further damage basal keratinocytes and hair follicle stem cells (16, 20, 21).

Currently, numerous medications are available for preventing ARD, but most cannot be recommended due to a low-quality evidence, a lack of supporting data, or conflicting findings across multiple trials (9). Fullerene is composed entirely of carbon atoms, which form hollow spheres resembling a football. These structures have conjugated double bonds, high electron affinity, and polarity, giving them an excellent ability to scavenge free radicals (22, 23). Research has indicated that fullerene can scavenge ROS and reactive nitrogen species (RNS) due to its special structure (24). Moreover, fullerene is more efficient in scavenging free radicals than conventional antioxidants, such as vitamin E or superoxide dismutase (SOD) (16, 25). The study (17) has demonstrated the radioprotective properties of fullerene as skin radioprotective agent in animal experiments. Thus, fullerene holds potentially radioprotective agent against ionizing radiation.

To the best of our knowledge, this is the first clinical study to investigate the prophylactic effect of fullerene on ARD. Our results demonstrate that EOSSKY Fullerene Moisturizing and Repairing Cream effectively reduces the grading and incidence of ARD in breast cancer patients undergoing radiotherapy. Furthermore, the cumulative dose of ionizing radiation at the first occurrence of ARD was significantly higher in the fullerene group compared to the control group, and the first appearance of ARD did occur later than in the control group. This may be attributed to fullerene’s ability to not only effectively scavenge various ROS, but also to induce endogenous phase II antioxidant enzymes and modulate cell antioxidant status by upregulating the antioxidant pathways in response to oxidative stress (26).

At the secondary endpoint, the fullerene group reported less pain. Additionally, according to the results of the Skindex-16, the fullerene group showed a significantly improvement in the patients’ QoL after radiotherapy. All of these findings suggest that EOSSKY Fullerene Moisturizing and Repairing Cream provides significant benefits in preventing ARD following radiotherapy for breast cancer.

However, this study has some limitations. First, the sample size was relatively small. Second, due to the different properties of EOSSKY Fullerene Moisturizing and Repairing Cream and the control intervention, it was not possible to blind physicians or patients, which may have introduce bias into the results. Third, although preclinical data and our clinical trials have demonstrated its safety and effectiveness, its long-term efficacy still requires further evaluation. Fourth, this study did not have biological evidence to confirm the antioxidant mechanism of fullerenes in patients due to resource constraints and ethical considerations, and future studies are recommended to bridge this translational gap.

## 5 Conclusion

In summary, this study found that EOSSKY Fullerene Moisturizing and Repairing Cream reduced grade and incidence of ARD, delayed the onset of skin reactions, alleviated pain, and significantly improved the patients’ QoL. These findings have important implications for the prevention of ARD in breast cancer and provides a valuable foundation for future research.

## Data Availability

The raw data supporting the conclusions of this article will be made available by the authors, without undue reservation.
